# UV-Light Curing of 3D Printing Inks from Vegetable Oils for Stereolithography

**DOI:** 10.3390/polym13081195

**Published:** 2021-04-07

**Authors:** Anda Barkane, Oskars Platnieks, Maksims Jurinovs, Sigita Kasetaite, Jolita Ostrauskaite, Sergejs Gaidukovs, Youssef Habibi

**Affiliations:** 1 Faculty of Materials Science and Applied Chemistry, Institute of Polymer Materials, Riga Technical University, P. Valdena 3/7, LV-1048 Riga, Latvia; Anda.Barkane@rtu.lv (A.B.); Oskars.Platnieks_1@rtu.lv (O.P.); Maksims.Jurinovs@rtu.lv (M.J.); 2 Department of Polymer Chemistry and Technology, Faculty of Chemical Technology, Kaunas University of Technology, Radvilenu Rd. 19, 50254 Kaunas, Lithuania; sigita.kasetaite@ktu.lt (S.K.); jolita.ostrauskaite@ktu.lt (J.O.); 3 Department of Materials Research and Technology (MRT), Luxembourg Institute of Science and Technology (LIST), 5 Avenue des Hauts-Fourneaux, L-4362 Esch-sur-Alzette, Luxembourg

**Keywords:** biopolymers, photopolymerization, kinetics, UV-light curing inks, additive manufacturing, stereolithography

## Abstract

Typical resins for UV-assisted additive manufacturing (AM) are prepared from petroleum-based materials and therefore do not contribute to the growing AM industry trend of converting to sustainable bio-based materials. To satisfy society and industry’s demand for sustainability, renewable feedstocks must be explored; unfortunately, there are not many options that are applicable to photopolymerization. Nevertheless, some vegetable oils can be modified to be suitable for UV-assisted AM technologies. In this work, extended study, through FTIR and photorheology measurements, of the UV-curing of epoxidized acrylate from soybean oil (AESO)-based formulations has been performed to better understand the photopolymerization process. The study demonstrates that the addition of appropriate functional comonomers like trimethylolpropane triacrylate (TMPTA) and the adjusting of the concentration of photoinitiator from 1% to 7% decrease the needed UV-irradiation time by up to 25%. Under optimized conditions, the optimal curing time was about 4 s, leading to a double bond conversion rate (DBC%) up to 80% and higher crosslinking density determined by the Flory–Rehner empirical approach. Thermal and mechanical properties were also investigated via TGA and DMA measurements that showed significant improvements of mechanical performances for all formulations. The properties were improved further upon the addition of the reactive diluents. After the thorough investigations, the prepared vegetable oil-based resin ink formulations containing reactive diluents were deemed suitable inks for UV-assisted AM, giving their appropriate viscosity. The validation was done by printing different objects with complex structures using a laser based stereolithography apparatus (SLA) printer.

## 1. Introduction

Under the so-called fourth industrial revolution, the interest in developing circular economies through the adoption of new manufacturing technologies and the substitution of fossil feedstock by renewable resources does not appear to wane but will continue to attract considerable attention in our modern society. Additive manufacturing (AM) or 3D printing is one of the most rapidly growing technologies, as it enables the manufacturing of objects with complex and advanced geometries that are usually difficult to achieve with other subtractive manufacturing techniques [1,2]. The manufacturing is achieved in a cost effective manner, as the consumption of materials is highly reduced by limiting its wastage, in contrast to conventional processes where the materials are removed from the bulk by milling, generating an important amount of waste [3]. The 3D structures are created by joining materials layer-by-layer using computer aided design. Although many technological variants have been reported, all of them fall under three main printing approaches, namely, fused deposition modeling, binder jetting or laser sintering/melting, and photopolymerization-based approaches. Thus, this highly versatile processing technique can be applied to polymer/plastic, metal, ceramic, concrete, and other materials. There are different techniques available, and the three basic requirements are the digital design, the 3D print technology, and the material used. In the case of polymer materials, the main AM technologies are fused filament fabrication/fused deposition modeling (FFF/FDM), selective laser sintering (SLS), laminated object manufacturing (LOM), stereolithography apparatus (SLA), etc. [4,5]. The latter technology was developed and then patented by Charles (Chuck) Hull et al. in 1984. SLA is a laser-assisted printing technique, and it is based on photopolymerization, a process in which a UV light or laser is directed in a pattern over a path of photopolymerizable liquid monomer or polymer, allowing the cross-linking of the liquid into a hardened layer. As each layer is polymerized, the printing platform can be lowered further into the polymer solution, allowing for multiple cycles to form a 3D structure [6]. Moreover, SLA can produce a vast number of highly different 3D structures in a reproducible way with precise control over the final microstructure and geometry. Yet, the main drawbacks to using SLA are the long post-processing step often required and the relative few materials compatible for use with SLA. Indeed, curable epoxies and acrylates liquid materials are the most used photopolymerizable material in SLA because of their high photoreactivity, which results in a high degree of fabrication accuracy. To overcome the material limitation, many efforts are devoted toward the development of novel UV-curable resins suitable for SLA technique. In this context, renewable building blocks derived from biomass are considered as attractive and inexhaustible starting materials for AM in general and for SLA in particular. Biomass is not only abundant, renewable, and sustainable, but also represents limitless potential to supplant or at minimum complement fossil fuel resources. Therefore, and in conjuncture with AM development, the direct use or the conversion of renewable resources into suitable 3D printable materials is surfacing as a potential alternative to develop a fully sustainable solution [7]. Some of the most widely used renewable raw materials in AM include cellulose and other polysaccharides, lignin and its derivatives, gelatin, and other biobased polymers such as polylactide, etc. [5,7,8,9,10,11,12,13,14].

Vegetable oils are abundant, renewable, and inexpensive, and exhibit structural features that make them attractive and sustainable chemical platform for a wide range of intermediates and products including polymeric resins and composites materials [15,16]. Indeed, innovative technologies have been developed to convert these natural resources into novel monomers and polymers, some of which have already generated competitive industrial products with properties comparable to conventional petrochemical polymers [17,18,19]. These vegetable oil-based polymers find applications in coatings, adhesives, sealants, elastomers, and foams [20]. A wide variety of plant oils can be used, but given their large production, the main used ones are soybean oil, palm oil, and rapeseed oil. The presence of a large number of unsaturated bonds make them a versatile chemical platform that allow the introduction of novel functionalities expanding therefore their reactivity. For example, the epoxidation reaction has been used extensively to modify plant oils to produce polymerizable oxygenated compounds [21]. Acrylation of the plant oils yields highly reactive groups and additional oxygen atoms in structure, making them suitable for thermoset polymer production [17].

Being liquids with the possibility to be epoxidized, acrylated, or thiolated, vegetable oils are being made reactive under UV for many applications [22,23,24,25]. More particularly, these vegetable oil derivatives are being used, among other renewable feedstocks, as feeding materials for UV-assisted AM techniques [26]. A fully biobased ink composed of a mixture acrylated epoxidized soybean oil (AESO) and vanillin dimethacrylate (VDM) or vanillin diacrylate (VDA) at different ratios was 3D printed under UV without the use of any photoinitiator nor solvent. The use of ultrashort pulses by multiphoton absorption and avalanche induced the polymerization and cross-linking of the biobased resins without the need for any photoinitiator. Intriguingly neat pure AESO showed higher crosslinking rate and better thermal properties than that of AESO with VDM or VDA [27]. AESO was also converted, through a 3D laser printing technique, into smart and highly biocompatible scaffolds capable of supporting growth of multipotent human bone marrow mesenchymal stem cells. The study showed that the superficial structures of the cured AESO were significantly affected by laser frequency and printing speed [28].

More recently, Voet’s groups developed biobased photopolymer resins based on modified soybean oil suitable for stereolithography. Various photoresin formulations were made by up to 80% of biobased acrylated or methacrylated epoxidized soybean oils. The resin composition was optimized to achieve suitable low viscosity by adding different content of monofunctional diluent, namely tetrahydrofurfuryl methacrylate or isobornyl methacrylate. The SLA printed parts demonstrated complete layer fusion and accurate print quality, while their stiffness and toughness were tuned by varying the chemical composition or the number of reactive sites [29]. An innovative and easy to prototype process was developed by printing a bio-based resin derived from soybean oil using optical 3D printing (O3DP) as an efficient and low waste production AM technique. The printing is made without the use of any photoinitiator and able to print objects with good reproducibly at sub-micrometer accuracy [30].

Given the tremendous interest in using vegetable oil derivatives as the main component to develop UV printable inks, we undertake in the present study an in-depth investigation of formulations based on AESO alone or in combination with reactive diluents. The UV-curing of various formulations was scrutinized by FTIR and photorheology, and the thermal and mechanical properties of the cured materials were examined. After the optimization, the suitability of these formulations for UV-assisted printing was validated by printing different objects.

## 2. Materials and Methods

### 2.1. Materials

Acrylated epoxidized soybean oil (AESO) (specification: contains 3500–4500 ppm monomethyl ether hydroquinone as inhibitor, viscosity is 18,000–32,000 cps.) was purchased from Merck KGaA (Darmstadt, Germany). 1,6-hexanediol diacrylate-technical grade 80% (HDDA) (specification: purity of > 77.5%) and trimethylolpropane triacrylate (TMPTA) (specification: purity of > 70.00%, contains 500–750 ppm monomethyl ether hydroquinone as inhibitor) both were purchased from Merck KGaA (Darmstadt, Germany). 2,4,6-trimethylbenzoyldiphenylphosphine oxide (TPO) photoinitiator (PI) was obtained from Arkema Lambson (Wetherby, UK). All chemicals were used as received. Structures of all raw components displayed in Figure S1 were photoactive moieties of active (C=C) bonds have been circled by marking all possible sites for crosslinking reactions.

### 2.2. Methods

#### 2.2.1. Fourier-Transform Infrared Spectroscopy

FTIR-ATR spectra were collected on a Nicolet 6700 (Thermo Fisher Scientific, Waltham, MA, USA) at a resolution of 4 cm^−1^ from 500–4000 cm^−1^. Sixteen scans of every specimen were performed, and the average spectrum was recorded. For photopolymerization, kinetics analysis measurements were performed on films obtained by UV-irradiation 5.5 W UV-LED lamp with wavelength of 405 nm at 2.5 cm between the resin and the UV source.

#### 2.2.2. Thermogravimetric Analysis

The thermogravimetric analysis (TGA) was performed on a Mettler Toledo TG50 (Columbus, OH, USA) instrument. Samples of about 10 mg were heated under nitrogen atmosphere at 10 °C/min from 25 up to 750 °C. The material thermal stability was evaluated from the weight-loss heating curves. The weight loss at 5%, 10%, 30%, 50%, 70%, and 90% was calculated according to ASTM D3850 by using the Mettler original software.

#### 2.2.3. Photorheology

Photorheology measurements were performed with a MCR302 rheometer from Anton Paar (Graz, Austria) with a plate/plate measuring system a Peltier-controlled temperature chamber with the glass, and the top PP08 plates (diameter of 38 and 8 mm, accordingly). The measurement gap was set to 0.3 mm, and the resin samples were irradiated using a UV/VIS spot curing system, OmniCure S2000, Lumen Dynamics Group Inc. (Mississauga, ON, Canada). Irradiation was performed at room temperature in a wavelength of 250 to 450 nm through the glass plates. Measurements were performed using shear mode with a frequency of 10 Hz and 0.3% of strain, while UV/VIS onset was 30 s. Storage modulus G’, loss modulus G”, loss factor tanδ (tanδ = G”/G’), and complex viscosity η* were recorded during the real-time photorheometry measurement.

#### 2.2.4. Dynamic Mechanical Analysis

Dynamic mechanical analysis (DMA) was performed by Mettler Toledo SDTA861e dynamic mechanical analyzer (Columbus, OH, USA). Measurements were performed on UV-cured films (8.5 × 4 × 0.3 mm^3^) and storage, loss modulus, and damping factor were characterized, with force of 10 N, frequency 1 Hz, elongation 10 µm, and in temperature −70–100 °C, heating rate of 3 °C/min.

### 2.3. Resins Formulation and Curing

Eight different resin formulations were prepared based on neat AESO or AESO/HDDA/TMPTA mixtures with a constant weight ratio of 65/30/5. Both monomers (HDDA, TMPTA) were used as reactive diluents to adjust the viscosity to SLA processing.

TPO was used as photoinitiator and loaded at 1%, 3%, 5%, and 7% of the total amount of the formulated resins. It was dissolved in few drops of acetone for better homogeneity; then consecutively HDDA, TMPTA, and AESO were added to the solution and carefully mixed. The mixture was then placed in the dark to avoid an unwanted reaction and kept for complete evaporation of acetone. The curing of the resins was performed using a 5.5 W UV-LED lamp with wavelength of 405 nm at 2.5 cm between the resin and the UV source. The exposure time was varied from 0 to 10 s with a 2 s step. The formulations were coined as AESO-X for neat AESO and M-AESO-X for formulations containing both AESO and reactive diluent monomers. X stands for the percentage of TPO. For example, AESO-5 and M-AESO-5 refer to neat AESO loaded with 5% of TPO and M-AESO-5 for the formulation with 65%, 30%, and 5% of AESO, HDDA, and TMPTA, respectively, and with 5% of TPO.

### 2.4. 3D Printing of Resins

After the optimization of the formulation and curing, the resins were printed using Original Prusa SL1 equipped with a Curing and Washing Machine (CW1). Original Prusa SL1 is based on the MSLA printing process and uses a 5.5” LCD display with 25 W LED power input and a UV wavelength of 405 nm, while the CW1 is equipped with 4 UV LED strips with 405 nm wavelength and maximum power of 52.8 W. After the process optimization, the irradiation time for set at 35 s for first 10 layers to allow the object being printed to properly adhere to the printing bed. The consecutive layers were then printed at 7.5 s irradiation time and layer height of 0.5 mm. After the printing, the objects were washed by isopropanol to remove all remaining resin from their surfaces and dried for 5 min after being post-cured in CW1 for an additional 3 min.

## 3. Results and Discussion

### 3.1. Investigation of UV-Crosslinking Process

Soybean oil triglycerides contain mostly unsaturated fatty acids: linolenic acid (7–10%), linoleic acid (51%), and oleic acid (23%). It also contains some saturated fatty acids such as stearic acid (4%) and palmitic acid (10%). It is made epoxidized and then acrylated to lower its viscosity and increase its reactivity toward UV-irradiation to be suitable for the SLA technique as it was used in the present study. Furthermore, HDDA and TMPTA were used as reactive diluents in order to further adjust the viscosity and increase the crosslinking density. The amounts of these reactive diluents were established based on previous reports in order to maintain high bio-based AESO content. Indeed, previous studies have investigated the concentration of these reactive diluents and their effects on the performances of the final materials. TMPTA has been used in multiple cases where its concentration ranged from 2% to 50% [31,32,33]. As a trifunctional monomer, this monomer contributes to the increase of the crosslinking rate and density, but at too high concentration it can lead to unwanted stiffness [22,34,35]. HDDA as bifunctional linear monomer contributes to the increase of the mechanical properties [24,36]. The photoinitiator used in this study is TPO and belongs to the Norrish Type I PI [37], where absorption of visible and ultra-violet (UV) lights causes homolytic bondage cleavage and generates two highly reactive radical species, which then initiate the polymerization and irreversibly incorporate into the polymer matrix. In the present study, the ratio between AESO/TMPTA/HDDA was kept constant at 65/30/5, and the photoinitiator was loaded at different concentrations ranging from 1% to 7%. The formulations were then exposed to UV irradiation at various exposure times from 2 to 10 s. As there are many possible combinations in this system, the crosslinking can occur between the same molecules (a-a; b-b; c-c) and/or between different molecules (a-b; a-c; b-c; etc.) in more than one site of each molecule. Given the number of reactive sites, the later combination is supposed to be the most dominant. Moreover, AESO has five reactive sites, while TMPTA and HDDA have three and two reactive sites, respectively. Therefore, HDDA will more likely prolong the crosslinking of chains, which is expected to increase the elasticity of the material. On other hand, TMPTA with more reactive moieties will more likely increase the crosslinking speed and density [38].

**Figure 1:**
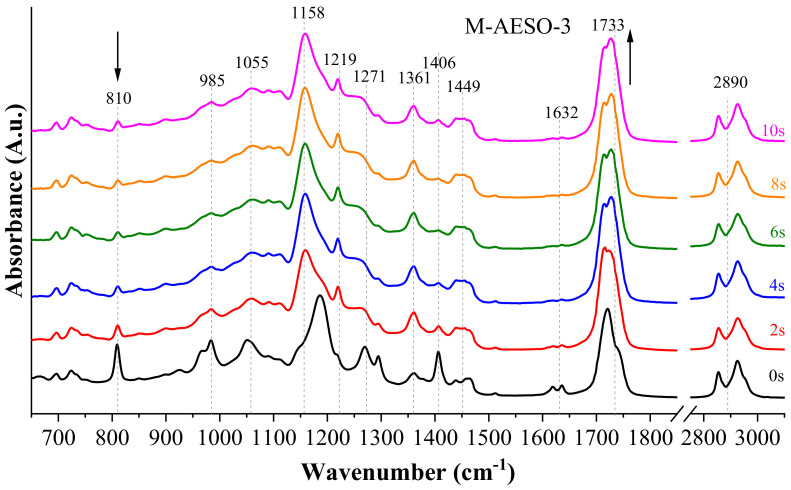
FT-IR spectra of AESO-3 (**top**) and M-AESO-3 (**bottom**) resins before and after UV curing at different exposure times.

The occurrence of the crosslinking was followed by FT-IR analyses for all formulations (neat AESO and M-AESO). In the Figure 1, the spectra of AESO-3 and M-AESO-3 are given as examples.

All characteristic peaks of groups present in AESO corresponding to (-OH) stretching vibrations of (C=O) and (C-O) ester groups are present before and after the UV exposure in all spectra at 3462, 1733 and 1219, and 1271 cm^−1^, respectively [39]. The bands corresponding to the asymmetric stretching of (O=C-O) at 1361 cm^−1^ [40,41] and stretching vibrations of ester (C-O-C) at 1158 cm^−1^ were also observed [39]. For AESO 3 resin, upon UV-irradiation, a decrease of the intensity is observed in (CH_2_=CH-R) scissioning and (C=C) at 1406 and 1632 cm^−1^, respectively. These decreases during the UV-irradiation happen because (C=C) bonds in acrylate groups are destroyed by the initiation of radicals and the occurrence of the crosslinking [36,40,42,43]. Additional conformation of acrylate polymer vinyl functionality is characterized by the (CH_2_=CH (CO)-O-) vinyl group at 985 cm^−1^, which also decreases upon UV-irradiation [40]. The decrease of the later peak affects also the peak at 1733 cm^−1^ related to the (C-O) of the ester groups, which is due to the electronic conjugation. Other characteristic peaks corresponding to asymmetric stretching vibrations and deformations of (C-H), (-CH_2_-), and (-CH_3_) groups were observed before and after the crosslinking at 1055, 2890, and 1449 cm^−1^, respectively. It is worth noting that no characteristic peak of the TPO was observed even at the highest loading, probably due to the low concentrations compared to the amount of the resins, but also because of the overlapping with the peak of the resins.

Before the characterization of the formulations containing the reactive diluents, FT-IR analyses of each of the used components before and after the UV-induced crosslinking were also performed to get preliminary insights on their interactions and crosslinking. The recorded spectra are provided in Figure S2. The spectra clearly show that both monomers are very similar from a chemical structure standpoint, but their crosslinking and physical properties aspects are different. Both reactive diluents as acrylates have similar characteristic peaks, also present in AESO, except for (-OH) band at 3462 cm^−1^, which is not present, since none of the monomers have this group in their formula. After the irradiation, the spectra of the monomers consist of much more irregularities in peak intensities than AESO. The intensity of (C=C) band at 810 cm^−1^ does not decrease as much; on the other hand, (O=C-O) at 1361 cm^−1^ decreases until complete disappearance for both reactive diluents, in contrast to AESO resins.

M-AESO formulations were then analyzed before and after the UV-curing at different exposure times. Similar characteristic peaks belonging to AESO, HDDA, and TMPTA were observed at different intensities depending on the curing times. One can observe a decrease of the peak corresponding to (CH_2_=CH (CO)-O-) vinyl group at 985 cm^−1^ during the irradiation, which was not complete as in the case of neat AESO resin, which is related to the higher amount of (C=C) bonds from acrylate groups brought by the addition of reactive diluents in M-AESO formulations than in neat AESO for all formulations. Similar observation applies to the peak at 810 cm^−1^.

**Figure 2:**
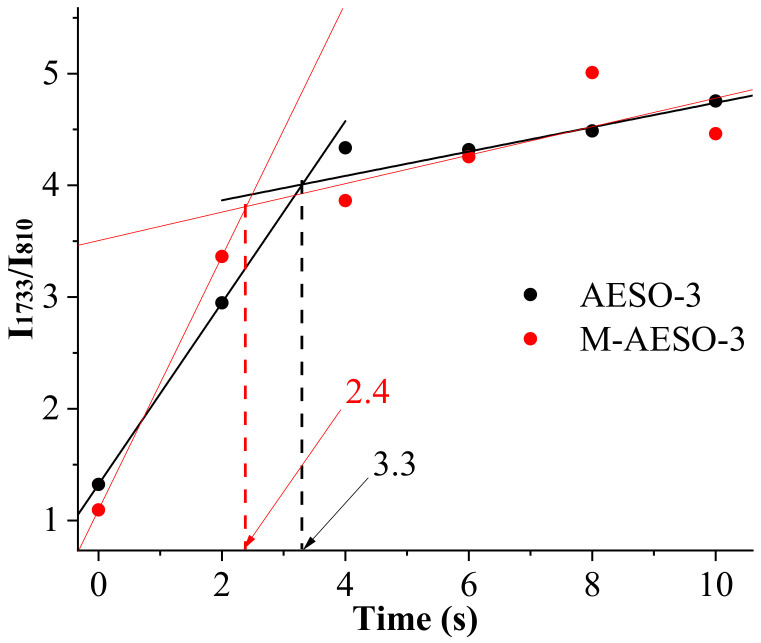
The variation of I_1733cm_^−1^/I_810cm_^−1^ with curing times of AESO and M-AESO resins at 3% loading of the photoinitiator.

FTIR measurements were used to evaluate the kinetic of the photopolymerization by following the intensities of the peaks at 810 and 1733 cm^−1^ corresponding to the (C=C) and (C=O) groups, respectively, for neat AESO resin and those containing reactive diluent M-AESO. The ratio of the two intensities I_1733cm_^−1^/I_810cm_^−1^ was used as the normalization means in order to eliminate possible errors that could arise from the differences in the thickness of the films. Figure 2 shows the evolution of the calculated ratio I_1733cm_^−1^/I_810cm_^−1^ against the curing times for the formulation, having 3% of TPO as an example. One can conclude that the onset optimal curing time is 3.3 s for AESO-3, while it is only about 2.4 s for M-AESO-3 resin, showing that the addition of the reactive diluents has increased the reactivity and hence decreasing the curing times. This applies to all loading of TPO. Indeed, the addition of the reactive diluents has decreased the curing time for all formulations regardless of the loading of the photoinitiator.

**Figure 3:**
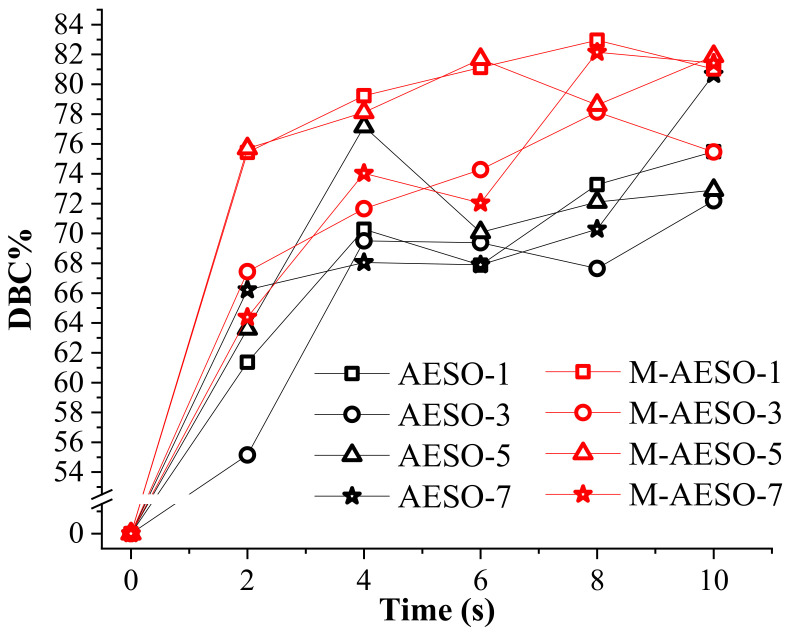
Double bond conversion (DBC%) rate as function of the UV-curing time for neat AESO and M-AESO for different loading of the photoinitiator.

The mechanical properties of the resulting polymer materials are significantly dependent on the crosslinking density and hence on the conversion of functional groups [44]. Therefore, double bond conversion rate (DBC%) was calculated from the ratio of the intensity of the peak at 810 cm^−1^ related (C=C) groups against the intensity of the peak at 1361 cm^−1^ corresponding to (COO^−^) groups, that is, unaffected by the photocrosslinking reaction, before and after the UV exposure at given time, using the Equation (1) [44,45]:

DBC% = (1 − (A_t_/A_ra_)/(A_0_/A_rb_)) × 100%, (1)

where A_t_ and A_0_—absorption intensities of the peak of (C=C) before and after UV-crosslinking and A_rb_ and A_ra_—absorption intensities of the peak of (COO^-^) before and after UV-crosslinking. Figure 3 depicts the variation of DBC% with curing time at different TPO loading, and the obtained curves confirm that the addition of polyfunctional comonomers increases the conversion degree by means of about 10%, as the values of DBC% obtained for M-AESO are higher than those obtained for neat AESO. DBC% for neat AESO reaches around 77% in 4 s in the case of 5% TPO loading, while it was slightly below for the other loading of TPO. After 4 s irradiation time, the DBC% remains constant or drops. This high conversion rate achieved under UV-curing attests for the fast reactivity of the acrylated moieties, because when compared to thermally cured dimethacrylate, the same value of conversion rate can be reached only after 3000 s of curing [46]. In the case of M-AESO, the DCB% increases sharply after only 2 sec of UV-irradiation and then continues to slowly increase after 4 s of UV irradiation, reaching 83% of conversion after 8 s. When compared to other vegetable oil-based resins reported in the literature about UV-curing, our prepared resin reached its highest conversion much faster. Indeed, in the case of acrylated epoxidized palm oil with no additional reactive diluents, DBC% was barely over 60% after 8 s [47]. It is worth noting that the increase of the amount of the photointiator does not automatically lead to higher absolute conversion degree. This could be explained by the screening effect, where the crosslinking starts rapidly at the upper layer, causing a complete absorption of the UV-light and consequently obstructing its in-depth penetration into the materials [48,49].

Another parameter of interest in the case of crosslinked network is the crosslinking density N and the related molecular weight between crosslinks M_c_. These two parameters were calculated according to the empirical approach adopted by Flory–Rehner [50,51,52]. The Table 1 gathers the calculated values of crosslinking density (N) and corresponding molecular weight between the crosslinks for all fully UV-cured resins (cured at 4 s). According to this rubber elasticity theory [53], the highest rigidity of the thermoset polymer is obtained for the polymer chain network with dense cross-linking structures and short distance between chain cross-links. Hence, the results show that in the case of the studied formulations, the UV-cured resins containing the functional monomers display almost five-fold enhanced crosslinking density characteristics in comparison to the cross-linked neat AESO resin. For example, M-AESO-3 resin formulation exhibits M_c_ = 45 g/mol and N = 73.9 × 10^3^ mol/cc in comparison to cured AESO-3 resin, which has only M_c_ of 193 g/mol and N of 16.4 × 10^3^ mol/cc, respectively.

**Figure 4:**
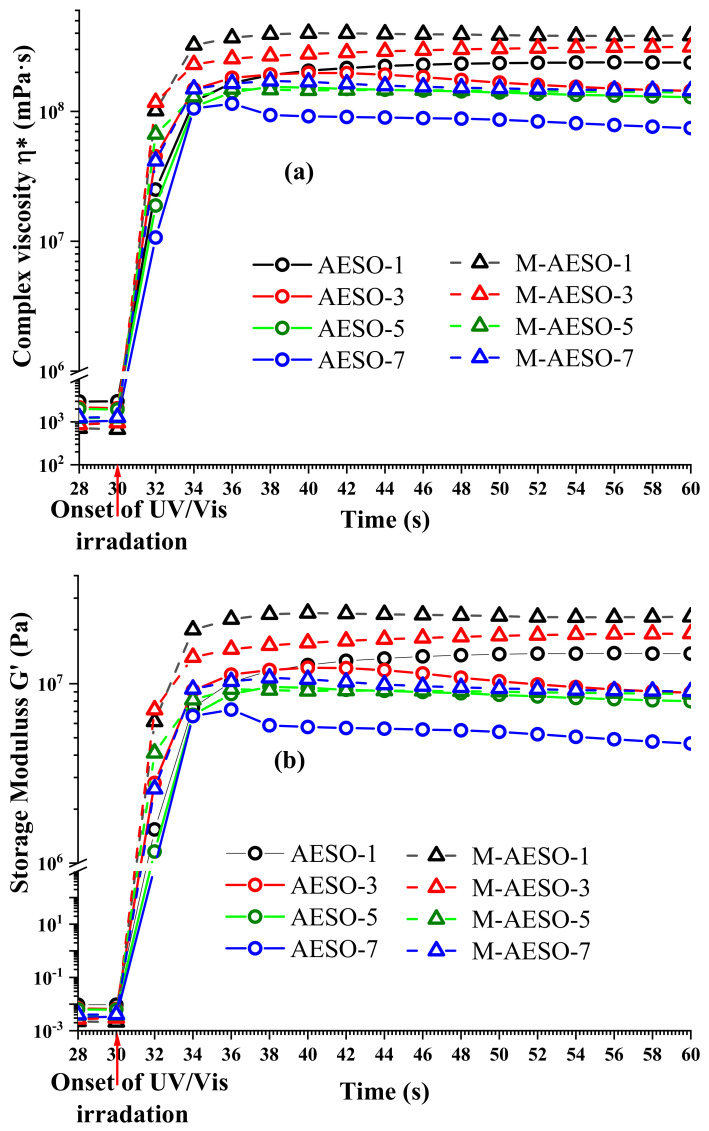
Complex viscosity η* (**a**) and Storage modulus G’ (**b**) photorheology curves for AESO and M-AESO resin formulations.

The photocuring of the different formulations was followed also by rheological measurements performed under UV-curing. Figure 4 shows the storage modulus G’ and the complex viscosity η^*^ photorheology curves of all AESO and M-AESO resins. Right after the onset of UV/VIS irradiation, the values of storage modulus G’ and complex viscosity η* start to increase very rapidly, attesting to the immediate formation of a three-dimensional polymer network, i.e., the start of chain cross-linking. At this point, their absolute value matches, and it is considered a gel point where high viscosity Newton liquid transforms into a hard-elastic polymer. The continuous slight increase of G’ is attributed to gel ageing and settling down into a steady-state that corresponds to continuation of the polymer chains crosslinking reactions [48]. After 4 s (34 s in the measurement curve) of UV-light irradiation, no changes in modulus and viscosity can be observed; thus, the final crosslinked network is considered to be formed [27]. Therefore, the curing time is determined to be around 4 s, which corroborates very well with the previous FTIR measurements for the same resin formulation.

Generally, the values of storage modulus G’ are directly related to the mechanical stability and crosslinking density of the polymer [27,54]. In Figure 4, these modulus values are laid out. Firstly, the loading of the monomers (M-AESO) has resulted in higher G’ (shear elastic moduli) and to a significantly steeper climb, which attest to an increase of the mechanical properties as well as the crosslinking rate upon the addition of the monomer. Secondly, by looking more closely, one can observe that increasing PI concentration results in a decrease of the modulus, although the absolute values of samples loaded with 5% and 7% of PI were very close. Indeed when a critical concentration of PI is exceeded, the effect of crosslinking is reduced as a result of limited penetration into the material because of rapid crosslinking of the surface layer; therefore, limited polymer crosslinking degree is achieved and the mechanical properties of the cured resins decreased [48]. Before irradiation, neat AESO resin formulations without functional monomers exhibit up to 55% higher complex viscosity than for the formulations M-AESO, with the highest being 2925 MPa·s for 1% of PI. This high viscosity recorded for neat AESO resins exceeds the range of the SLA printer, and therefore their printability could be compromised. After curing, e.g., when the crosslinking process is over, the M-AESO resin formulations with functional additives display even greater increase of the η^*^ than the neat AESO resin. Indeed, UV-cured M-AESO resins have 10 times higher η^*^ absolute values. In both formulations with or without reactive diluents, fully crosslinked polymer material has about a five-fold increase of complex viscosity compared to uncured resins. Moreover, at the same PI concentration, crosslinked samples show a greater increase in complex viscosity than uncured ones; the highest increase is 77%, and it was recorded for a sample with a PI concentration of 7%, as it exhibits after curing a viscosity value of 3.9 × 10^8^ MPa·s.

### 3.2. Thermomechnical Investigation of UV-Cured Materials

**Figure 5:**
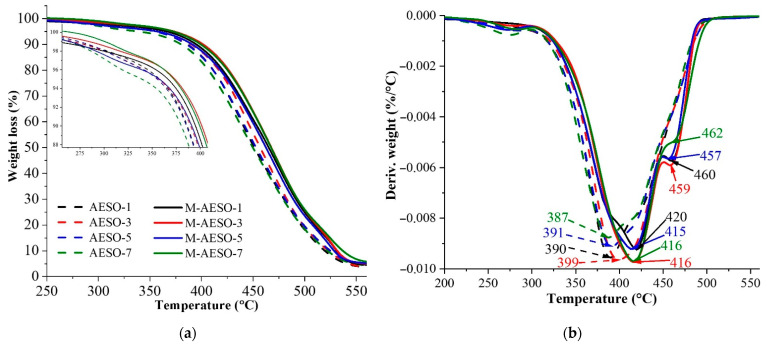
TGA thermograms (**a**) and the corresponding first derivatives TDG (**b**) of AESO and M-AESO resins formulations cured at 4 s.

In general, thermal and mechanical properties of a polymer network can be directly linked to the crosslink density [55]. After being cured, the resulting polymer materials were investigated in terms of their thermal and mechanical properties. The thermal stability is directly linked to the crosslinking chain network density, where higher degradation temperatures are characteristic of more dense and less defective polymer chain network [44]. Thermogravimetric analysis (TGA) was used to investigate the thermal stability of the cured materials. Figure 5a,b show the weight losses of the samples during the heating and the first derivative curve (DTG) T_max_ (temperature of maximums degradation rate), respectively. Both resins AESO and M-AESO exhibit closely similar degradation behaviors. Yet, the results show that the addition of the functional monomers in the case of M-AESO has increased the thermal stability by about 17–30 degrees for each formulation having the same concentration of PI. Indeed, the main degradation temperature ranges from 387 to 400 °C for neat AESO, while it was around 420 °C for M-AESO. Moreover, the presence of the monomer induced a second degradation of the samples at around 460 °C, which could be attributed to the degradation of added reactive diluents. This degradation behavior is likely to be attributed to TMPTA monomer, as has been suggested by other studies. Indeed, TMPTA monomer helps creating highly crosslinked density polymer network, which exhibits better heat-diffusion inhibition characteristics [36]. Table 2 gathers temperatures corresponding to different weight losses (from 5% to 90%). Overall, there is no significant weight loss until 250 °C (first 1% weight loss detected at 249 °C for sample M-AESO-1), and even at this temperature, the loss is very limited, as it represents only 1–2%, which could be related to the presence of incurable volatile components that degrade faster than cured network [27,47]. The main degradation takes place in the range of 300–500 °C, which is 50 °C higher compared to palm oil-based resin as reported in the Ashraf M. Salih et al. study [47]. Indeed, the main degradation of palm oil-based resin is reported between 250–550 °C. Additionally, 5% of weight is lost at 277 or 308.5 °C, depending on the used photoinitiator, while in our case, AESO, even without additional monomers, loses its first 5% at 339 °C, which is 30 °C higher compared to palm oil. Yet, in the present study, the char yields are 3–5%, while for palm oils, the char was around 2.3% [47]. The degradation results from random scission of the linear chains of the crosslinked system; therefore, branched systems with higher amounts of triacrylate monomer exhibit higher T_max_ values. A full investigation of the thermal stability of the produced resin inks was reported in our previous paper [56], where the kinetics of the thermal destruction were performed using Friedman method and revealed that the addition of the reactive comonomers increases the activation energy for thermal destruction by 10%.

**Figure 6:**
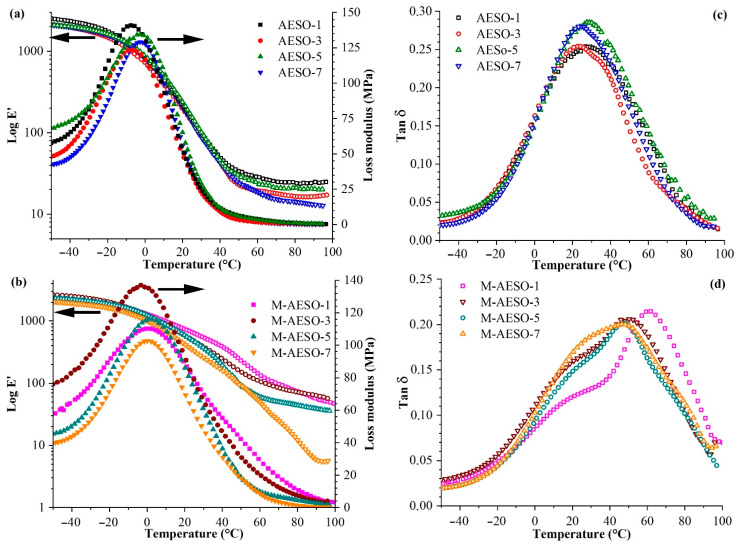
DMA curves for resin formulations cured at 4 s: (**a**) storage and loss modulus of AESO resins, (**c**) tan δ of AESO resins, (**b**) storage and loss modulus of M-AESO resins and (**d**) tan δ of M-AESO resins.

The mechanical properties of the studied resins were investigated by DMA measurements. Figure 6 depicts the obtained DMA curves for neat AESO and M-AESO resin formulations loaded with different PI concentrations and UV-cured at 4 s. Table 3 gathers the extracted values of the storage modulus at different temperatures and the glass transition temperatures. For both formulations, the DMA profiles are typical of thermoset resins where the storage modulus decreases upon heating the materials due to phase transition from a glassy state to a rubbery state. For thermoset resins, the storage and loss modulus values are a direct indication of the cross-linking density, which can be compared in either a rubbery (viscoelastic) state or at the glass transition region; yet, the glassy state is mostly indifferent to cross-linking density. In addition, it is well known that higher cross-linking density yields higher glass transition temperature, which is obtained from the dampening factor (tan δ) peak.

In the case of neat AESO resins, the storage modulus and loss moduli values are similar at the glassy state regardless of the concentration of the loading of the PI and thus the crosslinking density. The glass transition in this case occurs in a very narrow temperature window ranging between 24 and 29 °C, and the profiles show only one well defined peak for each formulation. At the rubbery state, there is a slight decrease of the values of the storage modulus upon the increase of the loading of the PI confirming the differences yet slight in the crosslinking density of the material. The sample with 5% initiator stands out with the best values of the storage modulus in the viscoelastic state and the highest T_g_, and a relatively small difference was obtained for the sample with 1% of added PI. The sample with 1% PI shows the highest loss modulus peak values and thus indicates a less stiff response, which is induced from AESO ability to self-plasticize; this synergy results in almost the same performance as other concentrations [57,58].

The same DMA profiles are observed in the case of formulations containing reactive diluents. The values recorded at the glassy state are comparable to those of the neat AESO, demonstrating as expected that at this state the crosslinking density has no effect over the values of the storage modulus. The M-AESO-3 exhibits the highest loss modulus value, while the formulation M-AESO-7 has the lowest loss modulus. The storage modulus values are strongly enhanced with the addition of the functional monomers by means of four-fold and five-fold increase at room temperature (20 °C) and higher temperatures (80 °C), respectively.

The glass transition occurs at higher temperatures compared to formulations without additives, attesting to a more rigid structure, hence increased crosslinking density. T_g_ values were 19–34 °C higher compared to those achieved for neat AESO samples. The dampening factor (tan δ) peaks of all formulations were broader than those obtained for neat AESO resins, exhibiting a shoulder for which the intensity increases with the decrease of the PI loading. The presence of this shoulder is indicative of the presence of phase separation. The occurrence of this phase separation is probably the reason of the higher loss modulus values recorded after the glass transition compared to other samples. The segmental mobility of loosely connected AESO sections could reduce overall stiffness of M-1 and M-3 samples, while this also seems to create better energy storing capabilities.

### 3.3. Printability of the Materials

**Figure 7:**
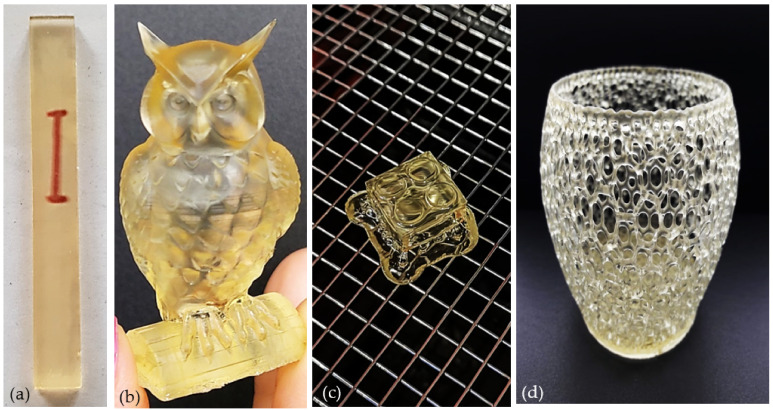
Printed M-AESO-3 resin, transparent bar (**a**), owl (**b**), a Lego cube (**c**), and basket like bowl (**d**).

After the optimization of the formulations and their UV-curing, the formulated resins were validated as UV-sensitive inks. Yet, given the high viscosity of the formulations based on neat AESO as determined by the rheological measurements, the printing of these resins was not possible with the used SLA printer, as it exceeded the operational range. Only formulations composed of AESO in combination with the reactive diluents were printable. Objects with more or less complex structures were then printed from all M-AESO formulations using SLA printers (Figure 7). These printed objects demonstrate complete layer fusion and accurate printing quality, as they were defect-free and exhibited high-resolution features and good mechanical properties, ensuring good stability without undergoing any shrinkage over the time.

## 4. Conclusions

Acrylated epoxidized soybean oil was used alone or in combination with reactive diluents as the main component to formulate UV-curable resins. The effect of the photoinitiator and UV exposure time were studied to understand the curability of these vegetable oil-based formulations. Optimal curing time to achieve full curing was about 4 s, as determined by photorheological measurements. The addition of the reactive diluents not only decreased the viscosity of the resins, but also induced a decrease in the photopolymerization time by 25% and an increase in the double bond conversion rate (DBC%) by 10%. Thermal degradation analysis showed good thermal stability of both types of formulations, which was improved by the addition of reactive diluents reaching about 420 °C as a consequence of the increased crosslinking density. The mechanical properties of the cured resins improved with the crosslinking density for the same type of formulations, which was related the content of the photoinitiator. Similarly, these properties were also improved upon the addition of the reactive diluents. Indeed, the storage modulus improved by almost four-fold at room temperature and five-fold at 80 °C, reaching 726 and 72 MPa, respectively, for the formulations containing the reactive diluents. Accordingly, the formulated sustainable bio-based resin inks containing the reactive diluents were deemed suitable for UV-assisted 3D printing, and thus they were successfully validated by printing different objects with complex structures with SLA printers. The formulated bio-based inks provided excellent printability showing a high promise for scalability toward commercial applications, yet further development and optimization are still needed.

## Figures and Tables

**Table 1:** Crosslinking density (N, mol/cc) and molecular weight (M_c_, g/mol) obtained for different formulations cured at 4 s.

Resin	M_c_ (g/mol)	N, ×10^3^ (mol/cc)
AESO-1	130	24.6
AESO-3	193	16.4
AESO-5	223	14.4
AESO-7	149	21.5
M-AESO-1	45	73.9
M-AESO-3	45	73.9
M-AESO-5	73	46.2
M-AESO-7	269	12.3

**Table 2:** Weight loss at thermal destruction.

Resin	T (°C) at Weight Losses						T_max_ (°C)
	5%	10%	30%	50%	70%	90%	
AESO-1	358	386	426	541	480	519	390
AESO-3	357	390	432	457	483	523	399
AESO-5	355	386	427	453	481	525	391
AESO-7	339	380	423	450	479	522	387
M-AESO-1	366	395	437	465	491	531	420
M-AESO-3	374	402	442	468	493	532	416
M-AESO-5	357	391	435	463	489	529	415
M-AESO-7	372	399	441	467	493	537	416

**Table 3:** Storage modulus values of AESO and M-AESO resin formulations cured at 4 s at different temperatures.

Resin	Storage Modulus (MPa)							T_g_ (°C)
	−40 °C	−20 °C	0 °C	20 °C	40 °C	60 °C	80 °C	
AESO-1	2337	1754	821	213	50	29	24	28
AESO-3	1970	1489	721	196	44	19	16	24
AESO-5	1996	1591	805	204	42	18	14	29
AESO-7	2016	1627	879	233	45	23	21	24
M-AESO-1	2273	1890	1262	726	385	136	72	62
M-AESO-3	2500	2014	1210	572	245	108	72	50
M-AESO-5	2280	1849	1241	572	194	60	45	48
M-AESO-7	1942	1622	1004	416	156	51	12	47

## Data Availability

The data that support the findings of this study are available from the corresponding author upon reasonable request. Research is still ongoing.

## Supplementary Materials

The following are available online at https://www.mdpi.com/article/10.3390/polym13081195/s1: Figure S1: Structure of AESO, monomer’s HDDA and TMPTA and photoinitiator TPO, Figure S2: FT-IR spectra of HDDA and TMPTA additives before and after curing at different UV-irradiation times.
