# Supplementary material for: UV-Light Curing of 3D Printing Inks from Vegetable Oils for Stereolithography
Source: Polymers (Basel). 2021 Apr 7;13(8):1195. doi: 10.3390/polym13081195 (PMC8068002; doi:10.3390/polym13081195)
Supplement: Supplementary file 1 [file polymers-13-01195-s001.pdf]

polymers

Supplementary Materials

UV-Light Curing of 3D Printing Inks from Vegetable Oils for Stereolithography

Anda Barkane [1], Oskars Platnieks [1], Maksims Jurinovs [1], Sigita Kasetaite [2], Jolita Ostrauskaite [2], Sergejs Gaidukovs [1]* and Youssef Habibi [3,]*

[1] Institute of Polymer Materials, Faculty of Materials Science and Applied Chemistry, Riga Technical University, P. Valdena 3/7, LV-1048 Riga, Latvia; Anda.Barkane@rtu.lv (A.B.); Oskars.Platnieks_1@rtu.lv (O.P.); Maksims.Jurinovs@rtu.lv (M.J.); Sergejs.Gaidukovs@rtu.lv (S.G.)

[2] Department of Polymer Chemistry and Technology, Kaunas University of Technology, Radvilenu Rd. 19, 50254 Kaunas, Lithuania; sigita.kasetaite@ktu.lt (S.K.); jolita.ostrauskaite@ktu.lt (J.O.)

[3] Department of Materials Research and Technology (MRT), Luxembourg Institute of Science and Technology (LIST), 5 avenue des Hauts-Fourneaux, L-4362 Esch-sur-Alzette, Luxembourg

* Correspondence: Sergejs.Gaidukovs@rtu.lv, Youssef.Habibi@list.lu

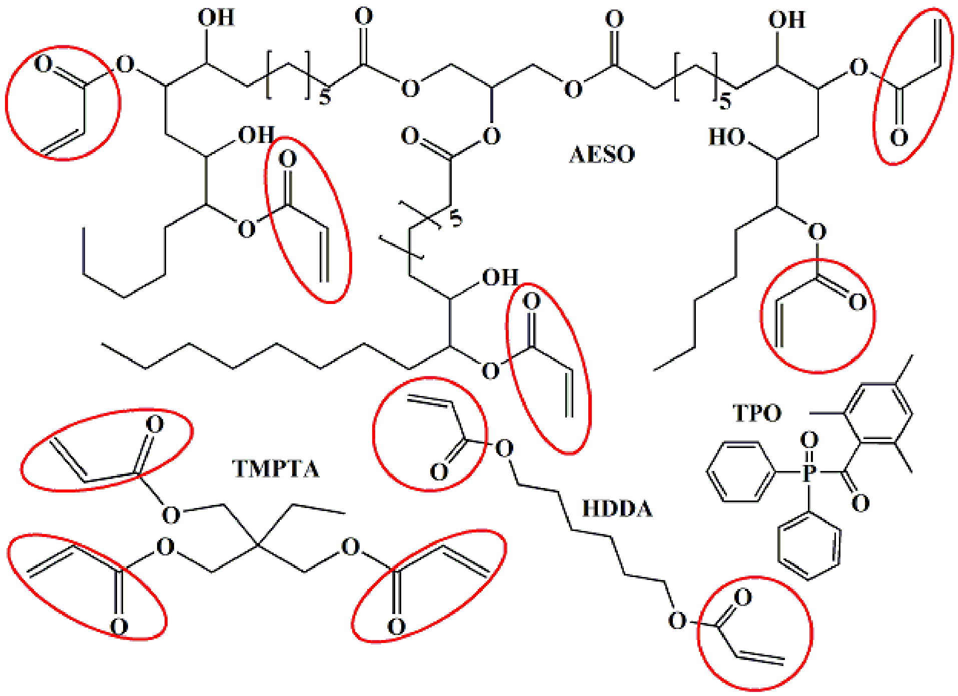

Figure S1. Structure of AESO, monomer's HDDA and TMPTA and photoinitiator TPO.

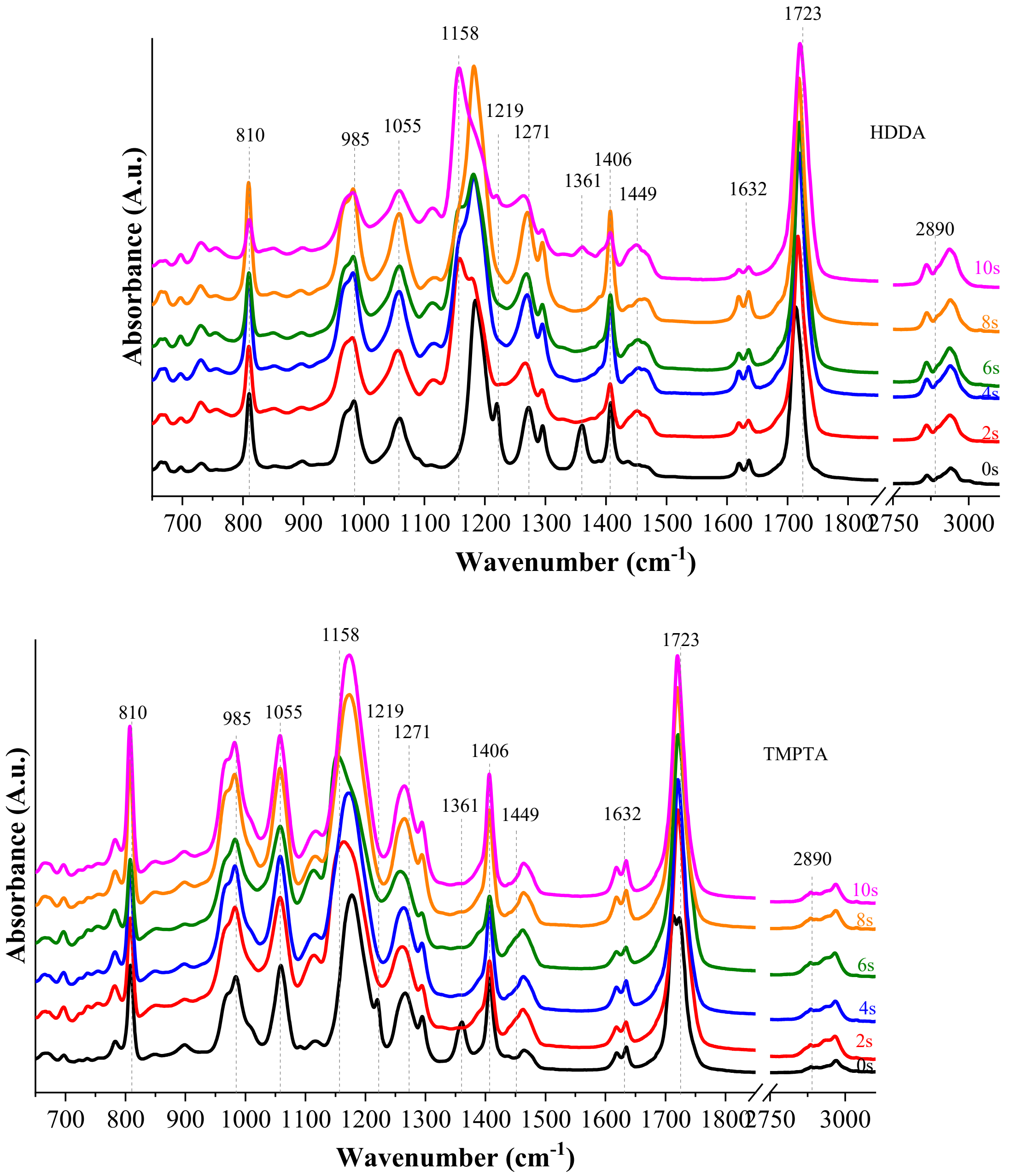

Figure S2. FT-IR spectra of HDDA and TMPTA additives before and after curing at different UV-irradiation times.
